# Supplementary figures and images for: Transcriptome Analysis Reveals Coexpression Networks and Hub Genes Involved in Papillae Development in Lilium auratum
Source: Int J Mol Sci. 2024 Feb 19;25(4):2436. doi: 10.3390/ijms25042436 (PMC10889295; doi:10.3390/ijms25042436)

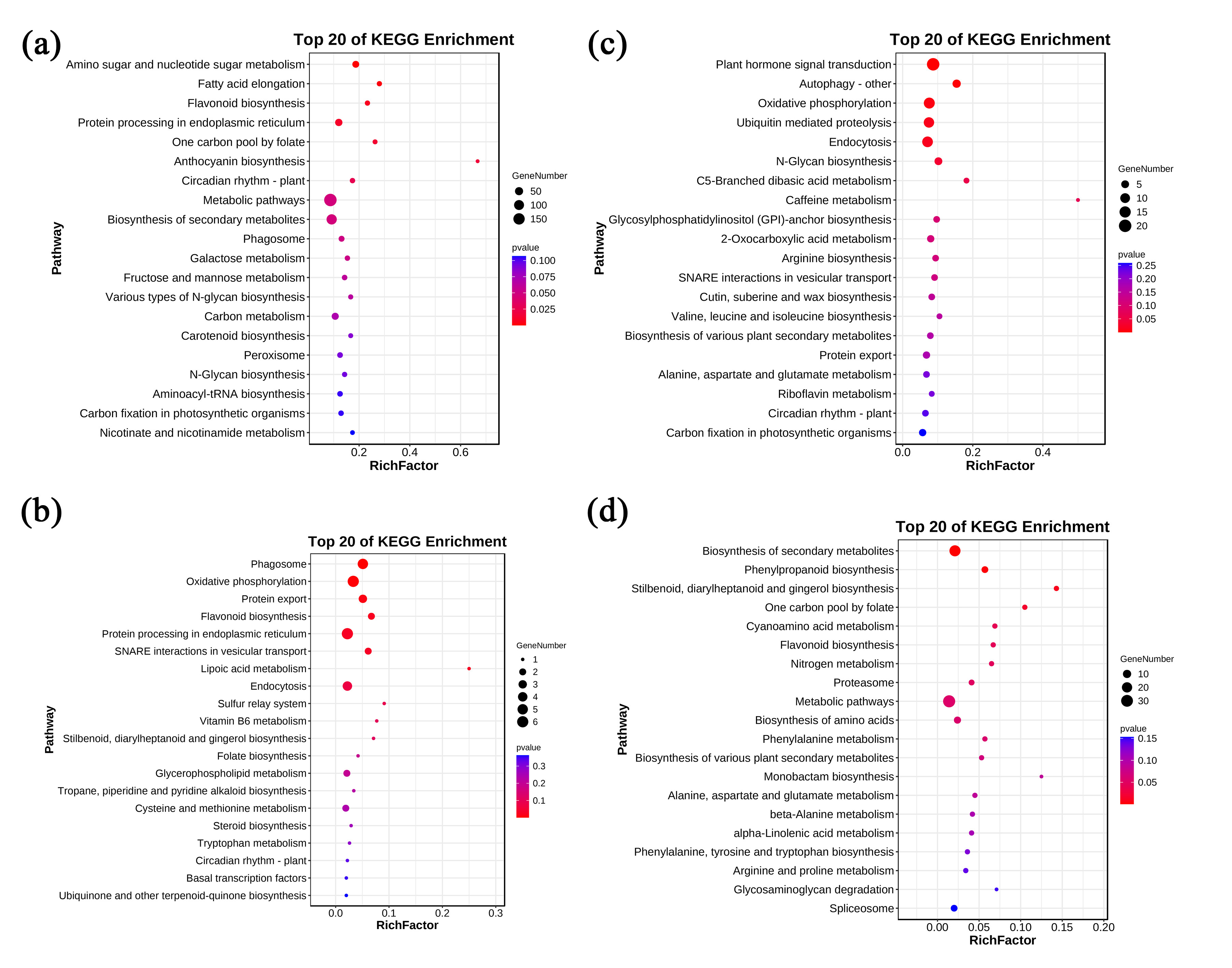

Supplement: Supplementary file 1 [file ijms-25-02436-s001.zip › Supplementary Figures/Figure. S2.jpg]

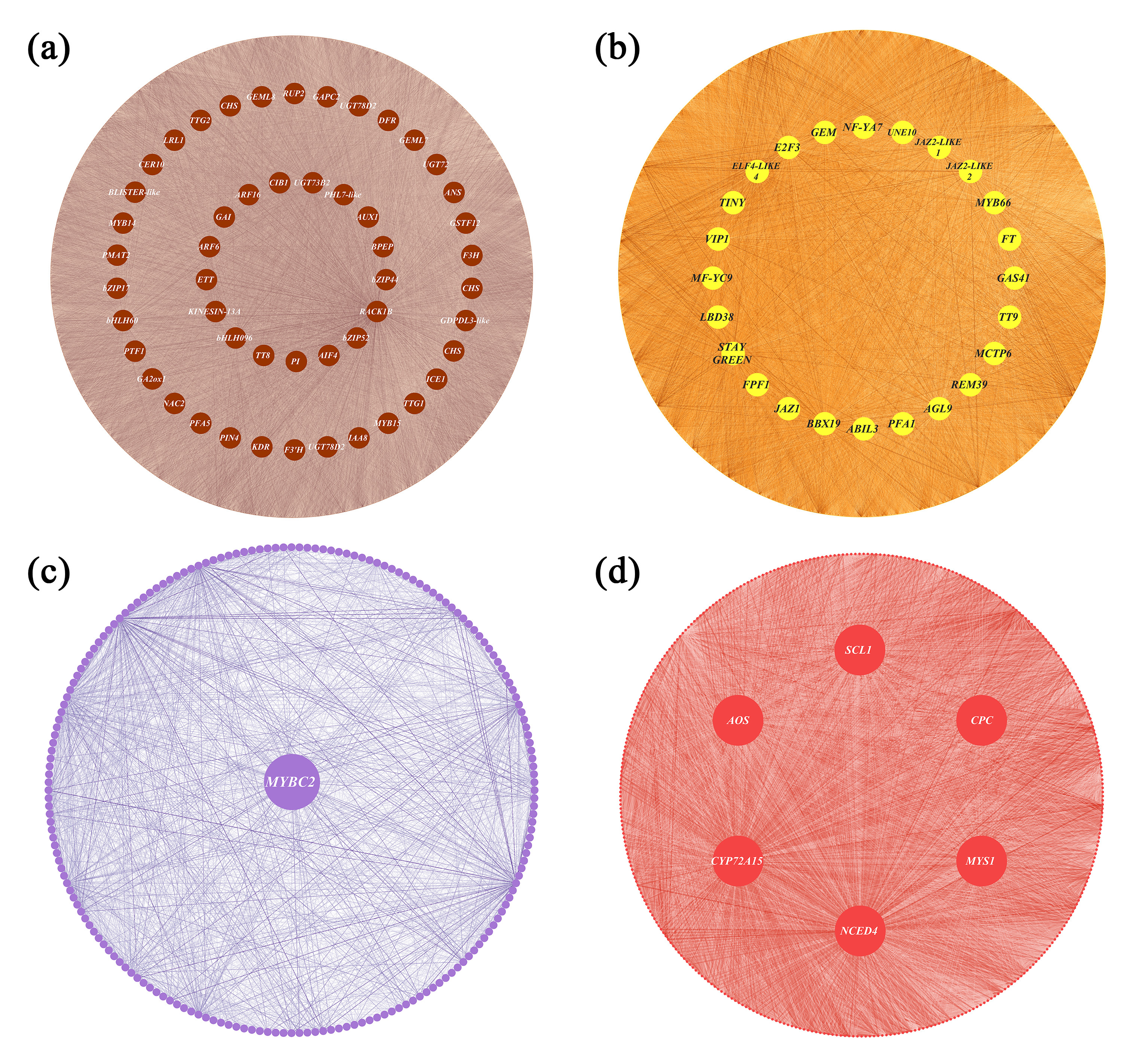

Supplement: Supplementary file 1 [file ijms-25-02436-s001.zip › Supplementary Figures/Figure. S3.jpg]

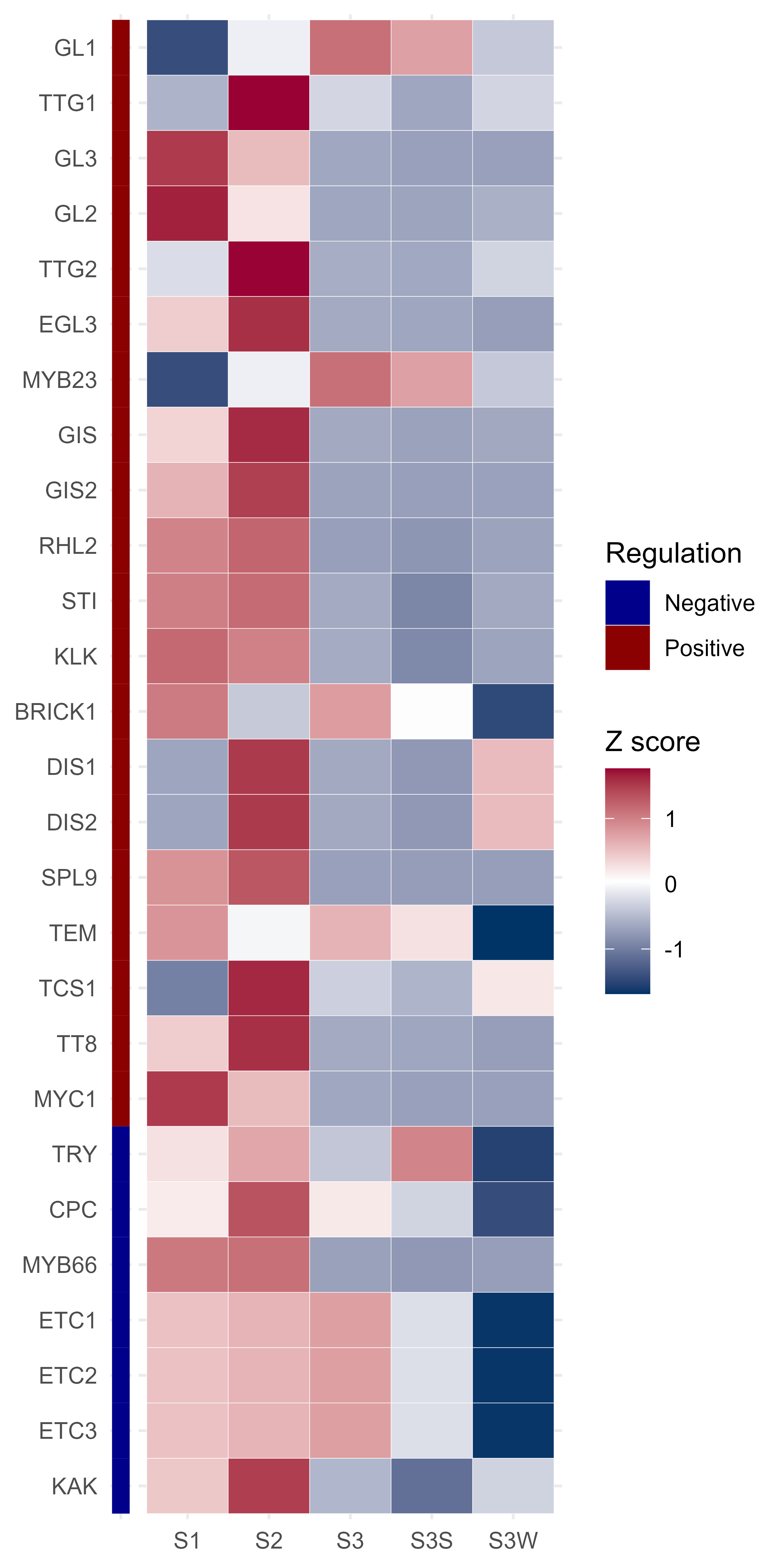

Supplement: Supplementary file 1 [file ijms-25-02436-s001.zip › Supplementary Figures/Figure. S4.jpg]

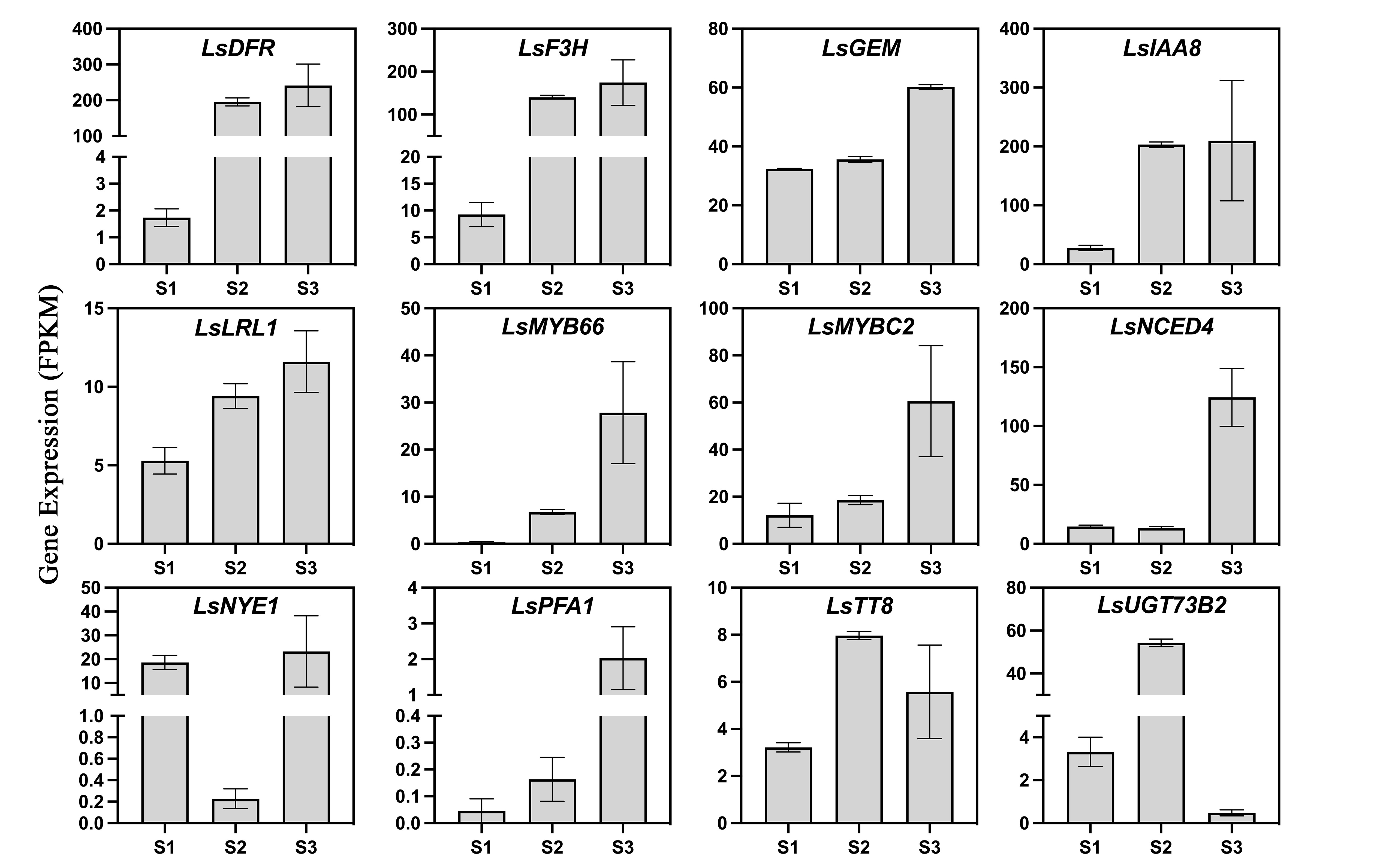

Supplement: Supplementary file 1 [file ijms-25-02436-s001.zip › Supplementary Figures/Figure. S5.jpg]

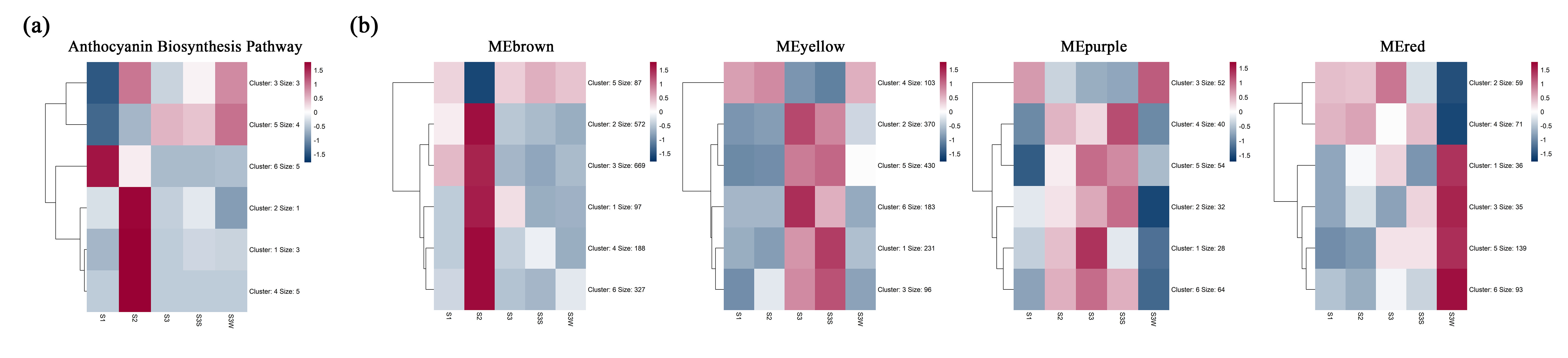

Supplement: Supplementary file 1 [file ijms-25-02436-s001.zip › Supplementary Figures/Figure. S1.jpg]
